# How emergency departments might alert for prehospital heat-related excess mortality?

**DOI:** 10.1186/cc5092

**Published:** 2006-11-10

**Authors:** Yann-Erick Claessens, Pierre Taupin, Gérald Kierzek, Jean-Louis Pourriat, Michel Baud, Christine Ginsburg, Jean-Philippe Jais, Eric Jougla, Bruno Riou, Jean-François Dhainaut, Paul Landais

**Affiliations:** 1 Paris Descartes University, Faculty of Medicine, Assistance Publique – Hôpitaux de Paris, Department of Emergency Medicine, Hôpital Cochin, 27 rue du Faubourg Saint-Jacques, F-75679 Paris Cedex 14, France; 2 Paris Descartes University, Faculty of Medicine, Assistance Publique – Hôpitaux de Paris, Department of Biostatistics, Hôpital Necker Enfants Malades, 149 rue de Sèvres, F-75007 Paris, France; 3 Paris Descartes University, Faculty of Medicine, Department of Emergency Medicine, Hôtel-Dieu, 1 place du Parvis Notre-Dame, F-75001 Paris, France; 4 Centre d'épidémiologie sur les causes médicales de décès, CepiDC Inserm, 78116 Le Vésinet, France; 5 Department of Emergency Medicine, CHU Pitié-Salpétrière, Assistance Publique Hôpitaux de Paris, Université Pierre et Marie Curie (Paris VI), boulevard de l'Hôpital, F-75013 Paris, France

## Abstract

**Introduction:**

A major issue raised by the public health consequences of a heat wave is the difficulty of detecting its direct consequences on patient outcome, particularly because of the delay in obtaining definitive mortality results. Since emergency department (ED) activity reflects the global increase of patients' health problems during this period, the profile of patients referred to EDs might be a basis to detect an excess mortality in the catchment area. Our objective was to develop a real-time surveillance model based on ED data to detect excessive heat-related mortality as early as possible.

**Methods:**

A day-to-day composite indicator was built using simple and easily obtainable variables related to patients referred to the ED during the 2003 heat-wave period. The design involved a derivation and validation study based on a real-time surveillance system of two EDs at Cochin Hospital and Hôtel-Dieu Hospital, Paris, France. The participants were 99,976 adult patients registered from 1 May to 30 September during 2001, 2002 and 2003. Among these participants, 3,297, 3,580 and 3,851 patients were referred to the EDs from 3 August to 19 August for 2001, 2002 and 2003, respectively. Variables retained for the indicator were selected using the receiver operating characteristic curve methodology and polynomial regression.

**Results:**

The indicator was composed of only three variables: the percentage of patients older than 70 years, the percentage of patients with body temperature above 39°C, and the percentage of patients admitted to or who died in the ED. The curve of the indicator with time appropriately fitted the overall mortality that occurred in the region of interest.

**Conclusion:**

A composite and simple index based on real-time surveillance was developed according to the profile of patients who visited the ED. It appeared suitable for determining the overall mortality in the corresponding region submitted to the 2003 heat wave. This index should help early warning of excessive mortality and monitoring the efficacy of public health interventions.

## Introduction

Unusually high temperatures with normal relative humidity were recorded during summer 2003 in Europe [1,2], especially in France [3]. Within a few days of the onset of the heat wave, the French National Institute for Public Health and Medical Research (INSERM) reported a sharp increase in the number of heat-related deaths [4]. Three hundred excess deaths were observed on 4 August 2003. These excess deaths progressively increased until 12 August, reaching 2,000 per day, and then rapidly stopped as soon as the temperature decreased after 14 August. Cumulative excess deaths, however, reached 14,800 over the first two weeks of August, corresponding to a 60% increase of expected mortality in France [4]. Heat-related illnesses were more frequent in elderly people during this period, especially those who lived in urban areas, as reported in previous heat waves [5]. Excess deaths gradually increased with age: from 120% for 50-year-old people to 170% for 85-year-old people [4]. The number of deaths at home and in nursing homes doubled during the heat-wave period.

The consequences of the heat wave were highest in the Paris area, with maximal temperatures measured above 35°C during 14 consecutive days from 1 August to 14 August 2003, including eight days with temperatures above 40°C and nine days with minimal temperatures above 35°C [6]. A 130% increase in the expected mortality was consequently observed. The Paris hospital administration (Assistance Publique – Hôpitaux de Paris) reported more than 2,600 excess emergency department (ED) visits, most of them classified as heat related, and reported 1,900 excess hospital admissions, unfortunately coinciding with a usual decrease of available beds during the summer period [7].

A major concern raised by the heat wave was the difficulty of rapid assessment of its direct consequences on patient outcome, mainly because of the delay in obtaining definitive mortality results. Previous studies reported that meteorological and real-time ED data can be useful to predict the occurrence of public health disorders [8]. Appropriate public health interventions, however, might decrease the effect of a heat wave; the prediction might then be distorted. We looked for an index that would detect overmortality even when the relationship between temperature and mortality changes.

Since ED activity reflected the overall increase of patients' health problems during this period, we attempted to develop a composite indicator to warn of excess mortality in the Paris area based on the profile of patients referred to the ED. We designed an observational study in order to describe the population admitted to the ED of two Paris teaching hospitals (Cochin Hospital and Hôtel-Dieu Hospital) for a period of five months (May-September) during 2001, 2002 and 2003, to develop a composite index that detects excess mortality using the data from the Cochin Hospital, and validate the index thus obtained on a validation set based upon the Hôtel-Dieu Hospital data.

## Methods

### Participants

Mortality data corresponded to the number of deaths per day in Paris over the study period. The index for heat-related excess mortality for use in emergency units was based on qualitative and quantitative variables registered in standard practice for patients admitted to the emergency room. These variables were daily patient flow, demographics (age, gender), medical (body temperature, heart rate, arterial blood pressure, oxygen haemoglobin saturation, blood glucose, respiratory rate), logistics (modality of ED referral: medical and nonmedical ambulances, taxi, firemen, police, self-referral) and patient outcome (discharge, hospitalisation in the intensive care unit, hospitalisation in a conventional medical unit, death in the ED).

Nurse triage using a four-level scale and the triage nurse diagnosis were also included. Triage nurse diagnoses contained 12 headings: respiratory features including dyspnea; chest pain and myocardial infarction; malaise; increased temperature; impaired general status; impaired consciousness; cardiovascular abnormalities (arrhythmia, hypo/hyper blood pressure); neuropsychiatric features; referral for social and legal purpose; abdominal and pelvic symptoms; trauma and joints symptoms; toxicology, dermatological or endocrine features and miscellaneous.

We studied day-to-day variations of the daily sample of subjects concerning these cited variables. The statistical unit used was the day. Patient consent was granted.

### Building the indicator: derivation study

#### Data

The indicator was built using a derivation sample, which consisted of the Cochin Hospital data: 52,773 patients were registered at the emergency room of the Cochin Hospital from 1 May to 30 September during 2001, 2002 and 2003. Data were routinely and prospectively captured into medical software used in these EDs (Urqual^®^; XR Partner, Paris, France). A specific application was designed to extract the relevant data.

#### Choosing the best percentage for a given variable

Quantitative variables (such as age or body temperature) were transformed into binary variables. For each quantitative variable, receiver operating characteristic curves were constructed to find the most appropriate threshold for heat-wave excess mortality. The threshold retained corresponded to the largest value of the area under the receiver operating characteristic curve.

A day was considered 'positive' for heat-wave-related excess mortality (the gold standard) if it belonged to the excess mortality period as described previously from 3 August to 19 August 2003 [4]. To neutralise a seasonal effect, a day-to-day adjustment was performed for the 2003 data by subtracting from the value of a given day in 2003 the mean of the corresponding day for 2001 and 2002. We retained the greatest value of the sum of specificity and sensitivity.

We then computed a percentage for each binary variable. For each day of the study, the numerator was defined as the number of ED patients presenting with the modality of the variable that was over-represented during the heat-wave period; the denominator was the number of ED patients.

For qualitative variables composed of more than two modalities, a similar day-to-day calculation was performed. The numerator was the sum of the counts of every modality over-represented during the heatstroke period, and the denominator was the number of ED patients.

The Paris Index of Heatstroke Related Excess Mortality (PIHREM) was composed of the product of selected percentages. Given the multiplicative structure of the indicator, we added 0.5 to the daily counts of each variable to avoid percentages equal to zero.

All combinations of percentages allowed building 2,047 indexes. The corresponding area under the curve (AUC) was computed for each index. The indices were then ranked according to their AUCs by increasing values and were labelled from 1 to 2,047.

The percentage of the presence of a given variable (for example age > 70 years) was represented using a local polynomial regression. On the abscissa we plotted the series of indices ranked according to increasing value of the AUCs. On the ordinate we plotted the estimate of the percentage of presence of the variable. We then selected the variables most frequently present in the best indices.

### Validation study

#### Data

The validation sample was based on the Hôtel-Dieu Hospital data: 46,996 patients from 1 May to 30 September during 2001, 2002 and 2003. Data were collected according to the same methodology as already described.

The retained index was calculated for the Hôtel-Dieu Hospital data. The validation index computed using the Hôtel-Dieu Hospital data was plotted and presented with the Paris mortality curve. The AUC for the Hôtel-Dieu index was also calculated.

### Pooled derivation and validation samples

The retained day-to-day indicator for heat-related excess mortality and the alert threshold was finally computed with the pooled data of Cochin Hospital and Hôtel-Dieu Hospital, and was plotted with the Paris mortality curve. The AUC for the index was then calculated.

An alert threshold was defined as the maximum value of the indicator outside the heat-related excess mortality period.

## Results

### Description of the patients

Data for 99,976 adult patients presenting to the two EDs were collected from 1 May to 30 September during 2001, 2002 and 2003 (Table 1). There was no significant difference between 2003 and years 2001 or 2002 in terms of ED registration numbers, age and sex.

During the same period, 225 patients (167 in Cochin Hospital, 58 in Hôtel-Dieu Hospital) were referred for heat-related conditions during the 2003 heat-wave period. The two samples differed for several characteristics. Patients from Cochin Hospital had more severe conditions as compared with patients from Hôtel-Dieu Hospital (data not shown). We recorded 71 cases of heat stroke at Cochin Hospital and 21 cases at Hôtel-Dieu Hospital; 18 patients admitted for heat-related conditions (7.7%) died in the EDs.

### Building the indicator: derivation study

#### Selecting percentages and building the best Cochin index

**Figure 1:**
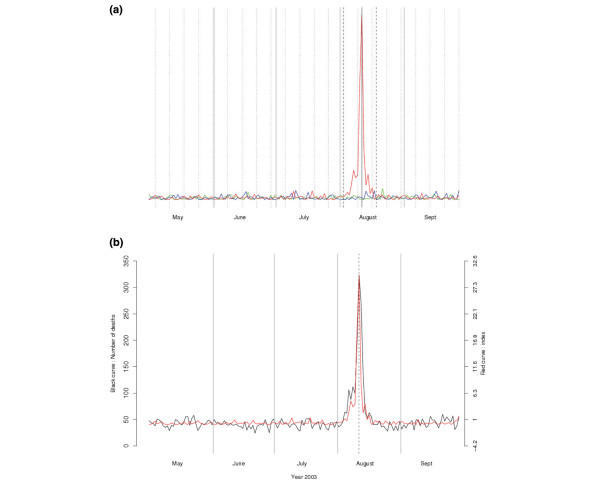
**Heatstroke-related overmortality indicator: derivation sample**. The three annual periods were superimposed and synchronised weekly. The vertical dashed lines define the heat-wave period (3–19 August 2003). **(a) **Best indicator calculated with the data of Cochin Hospital (2001, blue curve; 2002, green curve; 2003, red curve). **(b) **Cochin indicator (red curve) and Paris mortality (black curve).

The receiver operating characteristic curves of each variable and their AUCs were calculated. Using a local polynomial regression we represented the series of indices ranked by increasing value of AUCs. We thus selected the variables most frequently present in the best indices; namely, patients aged older than 70 years, patients with body temperature above 39°C, and patients admitted to or who died in the ED. The Cochin index is presented in Figure 1a,b.

### Validation study

#### Validation using the Hôtel-Dieu Hospital data

**Figure 2:**
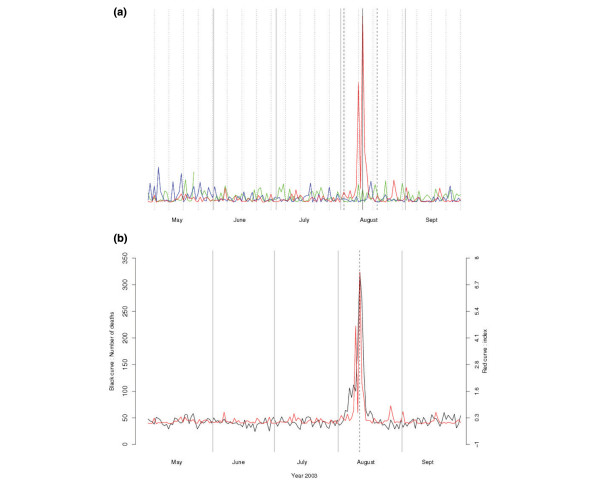
**Heatstroke-related overmortality indicator: validation sample**. The three annual periods were superimposed and synchronised weekly. The vertical dashed lines define the heat-wave period (3–19 August 2003). **(a) **Indicator calculated for validation using the Hôtel-Dieu Hospital data (2001, blue curve; 2002, green curve; 2003, red curve). **(b) **Hôtel-Dieu indicator (red curve) and Paris mortality (black curve).

The index calculated using the Cochin Hospital data was applied to the Hôtel-Dieu Hospital data (Figure 2a,b).

### The Paris Index of Heatstroke Related Excess Mortality

**Figure 3:**
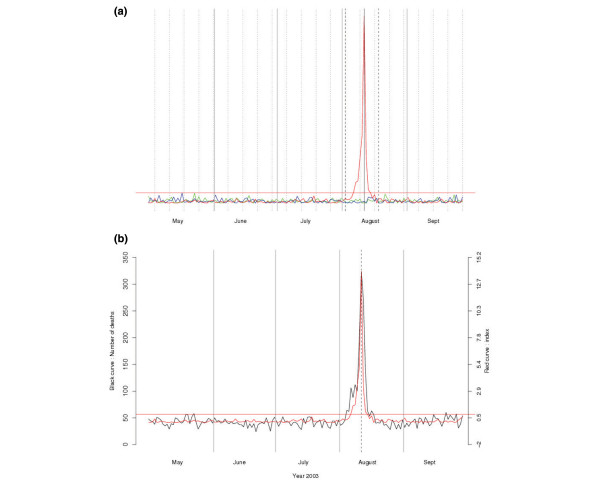
**The Paris Index of Heatstroke Related Excess Mortality**. The three annual periods were superimposed and synchronised weekly. The vertical dashed lines define the heat-wave period (3–19 August 2003). The alert threshold was defined as the maximum value of the Paris Index of Heatstroke Related Excess Mortality (PIHREM) outside the heat-wave period (horizontal red line). **(a) **Indicator calculated using the whole data (2001, blue curve; 2002, green curve; 2003, red curve) **(b) **The PIHREM (red curve) and Paris mortality (black curve).

The PIHREM was calculated using the pooled samples from Cochin Hospital and Hôtel-Dieu Hospital, and is shown in Figure 3a. The three annual periods were superposed and synchronisedby month. The index remained stable around a baseline – except during the heat-stroke excess mortality period, where it dramatically increased. The index appropriately matched the mortality, especially when it peaked on 12 August when most patients presenting with heat-related disorders died (Figure 3b). An alert threshold was defined as the maximum value of the indicator outside the heat-related excess mortality period, and appears on Figure 2a,b.

The threshold allowed alerting for excess mortality without latency. There was no false alert on the three year period because the threshold was greater than the maximum value of the indicator outside the heat-wave period.

## Discussion

Our study suggests that a simple index developed from characteristics of patients referred to EDs provides an immediate warning of an increased mortality rate during a heat-wave period based on a sample extracted from an urban area. The statistical model used variables that are easy to capture; namely, the number of patients older than 70 years of age with a central temperature above 39°C at admission who were admitted after an ED visit or who died in the ED. The association of these items strongly characterised the population referred to the EDs during the heat-wave period. More interestingly, it also coincided with the peak of mortality in the Paris basin.

Remarkably, the number of admissions increased only moderately during the heat-wave period compared with the corresponding periods of former years as reported by the Paris Hospital Administration [9]. The number of patients who visited the Cochin Hospital and Hôtel-Dieu Hospital EDs during the heat-wave period was significantly higher compared with previous years. It seemed more linked to an annual tendency than to an effect related to the heat-wave period itself. The number of daily visits therefore did not appear a relevant marker to alert emergency doctors in order to anticipate an excess mortality. The type of patients visiting the EDs, however, was different from other periods; we registered an increased number of patients who developed heat-related disorders and who needed specific care and hospital admission. This included heat stroke, but most cases corresponded to heat exhaustion.

Interestingly, characteristics of the population recruited in the two EDs during the heat-wave period differed for age, sex ratio, use of drugs that may promote heat-related disorder, mean temperature and number of patients with temperature above 39°C, and, finally, the number of patients with heat-related disorders and the number of patients requiring admission after an ED visit. This strengthens the validation of the PIHREM as this indicator can efficiently warn of excess heat-related mortality in two distinct samples.

As suggested in previous studies including results from the Paris heat wave, the city-related 'heat island' effect [10] should be considered a major risk for patients in developing heat-related conditions [11]. This is a matter of concern as most patients now live in urban areas. During the study period, patients admitted to EDs were more likely to develop hyperthermia and heat exhaustion [9,12] and to die from heat stroke despite hospital referral [13]. As a continuum exists between these case definitions, it was assumed that the excess of death during the heat-wave period could be attributed to heat-related organ failure [14].

Several studies have tried to detect the influence of social conditions, patient characteristics and their underlying disorders as risk factors for death related to abnormal heat. All of the reports on heat stroke agree on the influence of ageing. Our data also support the burden of the elderly on the occurrence of heat-related disorders, with a threshold of 70 years of age. Additionally, an underprivileged social environment [15,16], isolation [16] and independence in activities of daily living [17] have also been associated with the occurrence of heat-related death. Our study was not designed to evaluate these variables that are usually linked with ageing [16]. In studies from the United States, the impact of domestic characteristics was clearly determined [18,19]. Cardiac disorders such as heart failure and myocardial infarction were more frequent during the heat-wave period [17,20,21], sometimes combined with the use of psychotropic medication [17]. Local conditions may have an important influence on the occurrence of heat-related disorders. The level of sulphur dioxide combined with temperatures above 30°C was associated with an increased mortality rate in Athens, Greece. This was not specifically addressed in our study but may be of interest since heat-wave mortality increases in urban areas with increasing high air-pollution levels [22].

Patients who visited the EDs for heat-related conditions were mostly suffering heat exhaustion, with physical and laboratory parameters within normal ranges for most of them. We only recorded 18 deaths in the EDs. The number of deaths registered at home during this period was high – most of the frailest patients having died before hospital referral and most critically ill patients having been directly admitted to intensive care units.

Health authorities did not collect any information from EDs during the heat wave. An early warning might have modified management strategies that are key concerns for public health. French mortality data are currently collected retrospectively by the INSERM and published annually. Data collection comes from death certificates filed by medical practitioners. Consequently, the delay between overmortality and its detection impedes immediate reactivity.

Previous studies have reported that biometeorology can be a useful tool to predict the occurrence of public health disorders. For instance, changing weather is known to be a detrimental factor for the occurrence of myocardial infarction, especially in the case of modification of atmosphere fluxes, with a significant percentage of successful predictions [23]. Appropriate public health interventions, however, might change the impact of a heat wave on the population. Then, for given weather, the consequences on mortality might change. The results of a prediction model might therefore be modified. On the contrary, our index was fitted to detect an overmortality even when the relationship between temperature and mortality changes.

One of the limits raised by the PIHREM, however, is that it is unable to anticipate excess mortality. The PIHREM is not a predicting tool. It has been designed as a day-to-day detector of excessive mortality, not affected by public health interventions. It can be used as a tool to alert authorities.

Furthermore, EDs should be able to detect other events fraught with public health consequences, related to various risks, known or unknown; for example, in the case of bioterrorism attacks [24] or influenza. As suggested previously, collection of real-time morbidity data from EDs could have predicted heat-related deaths [8]. No system was available in France before or during the 2003 heat wave. The PIHREM is an initiative to support the early-detection capabilities by promoting immediate acquisition of relevant data with almost real-time reporting. Age, body temperature or admissions are nonspecific variables that might be applied to other conditions. Further prospective or retrospective studies are necessary to validate the model within other contexts.

## Conclusion

This paper reports that a composite marker can detect excessive mortality in a given area. It is suggested that EDs could be considered privileged places for supporting immediate alert for macroscopic events, helping though public health decision-making. French mortality data are currently not available in real time. Data collection comes from death certificates filed by medical practitioners. The delay between overmortality and its detection consequently impedes public health intervention. The PIHREM may help prevent this.

Our index mixes age over 70 years, body core temperature above 39°C and admission after ED visit or death in the ED, which are variables rapidly available after patient referral. Finally, EDs appear valuable centres of control to alert for heat-related prehospital excess of mortality while deaths do not necessarily occur in the ED. Computer networks between the French National Institute for Public Health and Medical Research (INSERM) and EDs are currently being developed; such indices could be useful to optimise population healthcare and the influence of future meteorological events such as heat waves, which seem likely to be more frequent [25].

The 2003 heat-related excess mortality raised questions about public health alerts and survey from the EDs with networks that should be set up. The PIHREM is a good indicator for warning of excess mortality based on real-time surveillance data that are easy to collect. Use of the PIHREM and the development of other indexes for other conditions may provide cornerstone information to advise authorities in public health management [8,24,26].

## Key messages

• The PIRHEM is a day-to-day indicator that detects an excess of mortality in an urban population exposed to a heat wave.

• EDs may easily provide the three selected variables that are rapidly available from the patients referred: age over 70 years, body core temperature above 39°C and admission after an ED visit or death in the ED.

• EDs are valuable centres of control to alert for heat-related prehospital excess of mortality.

• The PIRHEM might help monitor the efficacy of prevention and advising authorities in public health management.

## Abbreviations

AUC = area under the curve; ED = emergency department; INSERM = French National Institute for Public Health and Medical Research; PIHREM = Paris Index of Heatstroke Related Excess Mortality.

## Competing interests

The authors declare that they have no competing interests.

## Authors' contributions

PT participated in the study design, statistical analysis, interpretation of the results and writing of the report. GK participated in the study design, data collection, interpretation of the results and writing of the report. MB and CG participated in the study design and data collection for the report. EJ participated in the mortality data analysis. J-PJ participated in the study design, statistical analysis and interpretation of the results. J-FD, J-LP and BR participated in the critical revision of the report. Y-EC was responsible for the initial concept of the study design, data collection, interpretation of the results and writing of the report. PL was responsible for the retained study design, supervised the execution of the study, data collection and data analysis, and participated in the writing of the report. All authors read and approved the final version of the manuscript.

## Figures and Tables

**Table 1:** Patients referred to Cochin Hospital and Hôtel-Dieu Hospital emergency departments during the three year follow-up period

	1 May-30 September			3 August-19 August		
	
	2001	2002	2003	2001	2002	2003
Number of patients	31,600	33,196	34,973	3,297	3,580	3,851
Age, mean (standard deviation) (years)	41.2 (18.9)	41.0 (18.6)	40.7 (18.9)	41.0 (18.3)	41.0 (18.8)	43.5 (21.1)
Male, *n *(%)	17,854 (56.5)	19,287 (58.1)	19,970 (57.1)	1,840 (55.8)	2,158 (60.3)	2,140 (56.1)
Emergency department self-referral, *n *(%)	19,086 (60.4)	20,714 (62.4)	22,628 (64.7)	2,163 (65.6)	2,334 (65.2)	2,418 (62.8)
Triage level, *n *(%)						
Level 1	1,138 (3.6)	1,029 (3.1)	1,714 (4.9)	96 (2.9)	100 (2.8)	245 (6.4)
Level 2	9,037 (28.6)	10,490 (31.6)	11,402 (32.6)	788 (23.9)	1,078 (30.1)	1,240 (32.2)
Level 3	18,360 (58.1)	18,158 (54.7)	17,311 (49.5)	1,951 (59.2)	1,887 (52.7)	1,878 (48.8)
Level 4	3,065 (9.7)	3,519 (10.6)	4,546 (13)	465 (14.1)	516 (14.4)	488 (12.7)
Trauma, *n *(%)	11,092 (35.1)	10,855 (32.7)	10,492 (30)	1,048 (31.8)	1,049 (29.3)	974 (25.3)
Deceased at the emergency department, *n*	19	35	41	4	7	18
